# Absence of Inhibin Alpha and Retinoblastoma Protein Leads to Early Sertoli Cell Dysfunction

**DOI:** 10.1371/journal.pone.0011797

**Published:** 2010-07-27

**Authors:** Roopa L. Nalam, Claudia Andreu-Vieyra, Martin M. Matzuk

**Affiliations:** 1 Department of Pathology and Immunology, Baylor College of Medicine, Houston, Texas, United States of America; 2 Department of Molecular and Cellular Biology, Baylor College of Medicine, Houston, Texas, United States of America; 3 Department of Molecular and Human Genetics, Baylor College of Medicine, Houston, Texas, United States of America; Institute of Zoology, Chinese Academy of Sciences, China

## Abstract

Sertoli cells, the support cells of mammalian spermatogenesis, are regulated by a number of nuclear factors and express retinoblastoma (RB) tumor suppressor protein. We hypothesized that RB is an important mediator of Sertoli cell tumorigenesis in inhibin α knockout (*Inha* KO) mice. In our previous mouse studies, we found that conditional knockout (cKO) of *Rb* in Sertoli cells caused progressive Sertoli cell dysfunction. Initially, loss of RB had no gross effect on Sertoli cell function as the mice were fertile with normal testis weights at 6 weeks of age, but by 10–14 weeks of age, mutant mice demonstrated severe Sertoli cell dysfunction and infertility. Although double knockout (dKO) of *Rb* and *Inha* did not result in exacerbation of the tumorigenic phenotype of *Inha*-null mice, we found that the dKO mice demonstrate an acceleration of Sertoli cell dysfunction compared to *Rb* cKO mice. Specifically, in contrast to *Rb* cKO mice, *Inha/Rb* dKO mice showed signs of Sertoli cell dysfunction as early as 4 weeks of age. These results demonstrate that RB is not essential for Sertoli cell tumorigenesis in *Inha* KO mice but that loss of *Inha* accelerates the infertility phenotype of *Rb* cKO mice.

## Introduction

Mammalian spermatogenesis is dependent on the proper functioning of Sertoli cells, the somatic support cells of germ cell maturation. During embryogenesis, immature Sertoli cells emerge at E10.5 and form the seminiferous tubules [1]. They continually replicate prenatally and postnatally and finally enter replicative senescence at P12–17 when they differentiate into their mature form [2]. During puberty, Sertoli cells must form extensive intercellular junctions to support an expanding population of differentiating germ cells undergoing the process of spermiogenesis, which involves extensive morphological differentiation of round spermatids into mature spermatozoa [1]. At 5–6 weeks of age, mice produce spermatozoa from the first wave of spermatogenesis, and previous studies suggest that this first wave of spermatogenesis is significantly different from later waves [3], [4], [5]. At this point, Sertoli cells must once again adapt because, unlike the first wave of spermatogenesis, subsequent waves of spermatogenesis not only depend on forming new cellular junctions but also depend on removing old ones [1]. Our previous work in this field indicated that retinoblastoma protein (RB) is essential to maintain cell cycle quiescence and junctional remodeling in mature adult Sertoli cells [6].

RB is an important intracellular regulator of the cell cycle. RB is a repressor of E2F transcription factors, which function to promote progression from G1 to the S phase of the cell cycle [7]. For the cell cycle to progress, RB is inactivated via phosphorylation by cyclin dependant kinases (CDKs). RB and RB pathway components are frequently altered in many human cancers [8], but little is known about the role of RB in Sertoli cell tumorigenesis.

Inhibins are α:β heterodimeric members of the transforming growth factor β (TGFβ) superfamily and act as competitive antagonists of activins, which are β:β homodimeric TGFβ ligands [9]. Targeted deletion of the inhibin α subunit (*Inha*) in mice revealed that loss of inhibin leads to the development of gonadal sex cord-stromal tumors, which originate from Sertoli cells in the male [10]. Further studies using *Inha*-null mice deficient in either follicle stimulating hormone (FSH) and/or luteinizing hormone (LH) revealed that these tumors were gonadotropin-dependent [11], [12], [13], but the intracellular regulators of Sertoli cell tumorigenesis remained unknown [14]. Subsequent studies showed that RB pathway components, cyclin D2 (*Ccnd2*) and cyclin dependant kinase 4 (*Cdk4*), are increased in *Inha* knockout (KO) tumors while the CDK inhibitor, p27 (*Cdkn1b*), is decreased [15]. Double knockout (dKO) studies in our laboratory revealed that p27 [15] and cyclin D2 [16] are important modifiers of the *Inha* KO phenotype as *Inha/Cdkn1b* dKOs had accelerated cancer development, whereas *Inha/Ccnd2* dKOs had attenuated tumor formation.

Based on these findings, we hypothesized that RB is essential for the progression of *Inha* KO tumorigenesis and, similar to *Inha/Cdkn1b* dKO, that *Inha/Rb* dKO mice would demonstrate rapid tumor formation. We endeavored to carefully characterize this double knockout of *Rb* and *Inha* in the male to better understand the role of RB in Sertoli cell cycle control and tumorigenesis. Unexpectedly, the loss of RB on an *Inha* deficient background does not worsen disease progression. However, our findings show that loss of inhibin accelerates Sertoli dysfunction in the Sertoli cell-specific RB knockout mice [6].

## Materials and Methods

### Mouse Lines and Genotyping

Generation of mice containing a null mutation in the *Inha* gene [10], a null [17] or floxed [18] mutation in the *Rb* gene, and a transgene of Cre recombinase driven by the anti-Müllerian hormone promoter (*Amh-Cre*) [19] have been described previously. Tail DNA was utilized for PCR genotyping that was performed for all alleles according to the manufacturer's protocol (New England Biolabs, Ipswich, MA). Primers for the *Inha* alleles have been described [20] (E2-2/Ex2: 5′-GGTCTCCTGCGGCTTTGCGC-3′; INTRON: 5′-CCTGGGTGGAGCAGGATATGG-3′; Hprt3: 5′-GGATATGCCCTTGACTATAATG-3′) and produce wild-type (550-bp) and null (850-bp) products. Primers for *Rb* exon 3 alleles have been described [6] (RX3: 5′-GCATCTGCATCTTTATCGCAG-3′; RI3.1: 5′-CACCTTAGGCCGGGCAGTG-3′; PGK: 5′-GAAGAACGAGATCAGCAGCC-3′) and produce wild-type (724-bp) and null (400-bp) products. Primers for *Rb* exon 19 alleles have been described [21] (Rb212: 5′-GAAAGGAAAGTCAGGGACATTGGG-3′; Rb18: 5′-GGCGTGTGCCATCAATG-3′) yielding a 748-bp product for the *Rb*-floxed allele, a 699-bp product for the *Rb* wild-type allele, and a 260-bp product for the recombined allele. PCR for *Amh-Cre* has been described [6] (McreAMH: 5′-AGCTCAGGCCTCTGCAGTTA-3′; McreGene: 5′-AATCGCGAACATCTTCAGGT-3′) and produces a 443-bp product.

### Animal Care and Treatment

Mice were maintained on a 129SvEv/C57BL/6 background and housed with unlimited access to food and water and exposure to 12 h∶12 h light:dark cycles in accordance with the standards of the Association for Assessment and Accreditation of Laboratory Animal Care. The Institutional Animal Care and Use Committee at the Baylor College of Medicine approved the study under approval number AN-716. Survival curves were generated as described [13]. Mice were weighed weekly from 4 weeks to a maximum of 26 weeks to monitor for symptoms of cancer cachexia and sacrificed when body weights decreased to less than 16.0 g. For serum collection, mice were anesthetized, and blood was collected by cardiac puncture. Microtainer tubes (BD) were utilized for serum isolation and sent to the University of Virginia Center for Research in Reproduction Ligand Assay and Analysis Core (http://www.healthsystem.virginia.edu/internet/crr) for detection of FSH, LH, testosterone (T), and estradiol (E2).

### Morphological and Histological Analysis

Immediately after cardiac puncture, mice were euthanized, and the desired tissues were harvested and weighed. In general, one testis/tumor was placed into fixative and the other was frozen for DNA (−20°C) and RNA (−80°C in RNA*later*, Qiagen) analysis. Tissues were fixed in 10% neutral buffered formalin (Harleco) or Bouin's fixative (Sigma) prior to paraffin embedding. Tissue embedding, sectioning, and staining for periodic acid Schiff and hematoxylin were performed by the Histology Core of the Department of Pathology of Baylor College of Medicine.

### RNA Extraction and Quantitative PCR

RNA was extracted from 4 week-old testes using the RNeasy Mini Kit (Qiagen, Valencia, CA). For quantitative PCR, the isolated RNA (n = 3 for each genotype) was converted to cDNA using SuperScriptIII (Invitrogen). Primers (*E2f1*, *Cdkn2a*, *Timp1*) used in a SYBR green based qPCR assay were previously reported [6], [22]. *Bbc3* was assayed with a Taqman probe (Mm00519268_m1, Applied Biosystems). Quantitative PCR reactions were performed using *Gapdh* as an endogenous control for relative quantification [22].

### Statistical Analysis

Statistical analysis utilized JMP 8.0.1 software (SAS Institute). Statistical significance was determined by one-tailed *t* test assuming unequal variance for two sample comparison and by one-way analysis of variance (ANOVA) followed by Tukey's honestly significant difference (HSD) test for multiple sample comparisons. Survival curves were compared using the log-rank test. Groups were considered not significantly different from one another if p>0.05.

## Results

### Loss of Sertoli cell-expressed RB does not adversely affect disease progression in *Inha*-null mice

**Figure 1:**
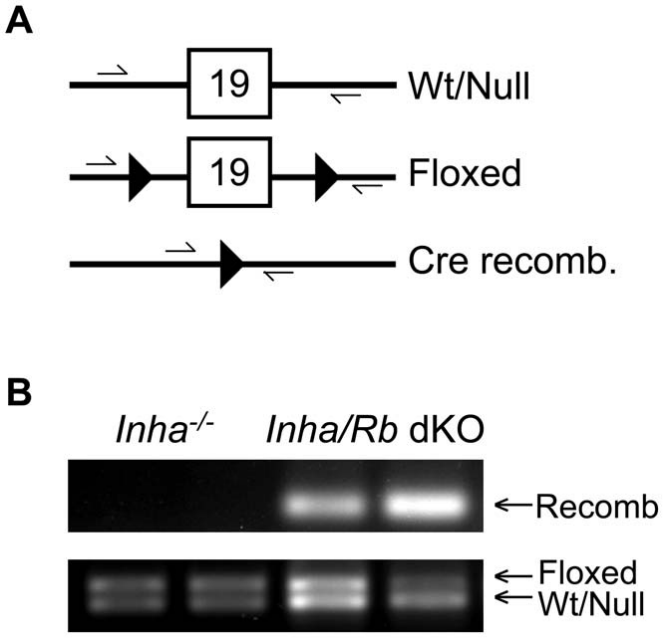
Proof of *Rb* recombination in testes of 6 week-old mice. PCR amplification of the region surrounding exon 19 was performed on DNA extracted from whole testis of *Inha^−/−^* and *Inha/Rb* dKO mice. As shown schematically in (A), primers flanking the loxP sites amplify the recombined *Rb* conditional allele to produce a 260-bp product, which is only seen in the Cre-positive mice (B). Amplifications of a 748-bp product for the *Rb*-floxed allele and a 699-bp product for the *Rb* wild-type/null allele were used as loading controls (B).

*Inha* KO mouse tumors arise from Sertoli cells or their precursors [10]. Since we wanted to delete RB from these tumors and *Rb*-null mice are embryonic lethal [17], we employed a conditional knockout (cKO) system that we had previously used to delete *Rb* in Sertoli cells [6] by utilizing a floxed *Rb* allele [18] and a Cre recombinase driven by the anti-Müllerian hormone (*Amh*) promoter [19]. Genotypes examined include: *Inha^+/−^ Rb^flox/−^* (control, *Inha^+/−^ Rb^+/−^*), *Inha^+/−^ Rb^flox/−^ Amh-Cre* (*Rb* cKO), *Inha^−/−^ Rb^flox/+^* (*Inha^−/−^*), and *Inha^−/−^ Rb^flox/−^ Amh-Cre* (*Inha/Rb* dKO). As shown in Figure 1, recombination was confirmed to occur in the dKO mice using 6 week-old whole testes.

**Figure 2:**
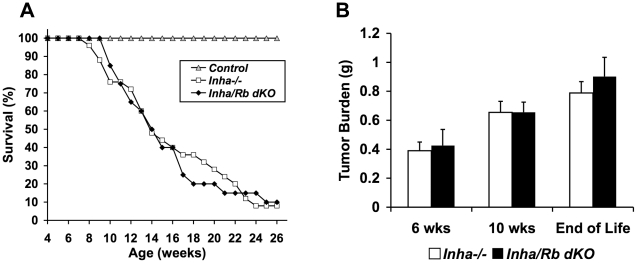
Double knockout of *Inha* and *Rb* in Sertoli cells does not significantly affect disease progression. Control mice have 100% survival until 26 weeks of age (A). During the same period, *Inha^−/−^* (n = 25) and *Inha/Rb* dKO (n = 20) mice reach 50% survival at 14 weeks of age and are not significantly different by log-rank test (A, p>0.05). Tumor burden is also not significantly different between these two groups at 6 weeks, 10 weeks, and end of life (B, p>0.05).

After we had confirmed that the *Rb* allele was recombined in our dKO mice, all groups of mice were weighed weekly starting at 4 weeks of age to monitor disease progression since *Inha* KO mice experience death secondary to cancer cachexia [23]. We monitored these groups until they reached 26 weeks of age and found that while 100% of control and *Rb* cKO mice survived until the end of the observation period, only 8% of *Inha^−/−^* and 10% of *Inha/Rb* dKO mice survived to that age (Figure 2A). The difference in mortality between *Inha^−/−^* and *Inha/Rb* dKO mice was not significant (p>0.05 by log-rank test), and both groups had 50% survival at 14 weeks of age (Figure 2A).

**Figure 3:**
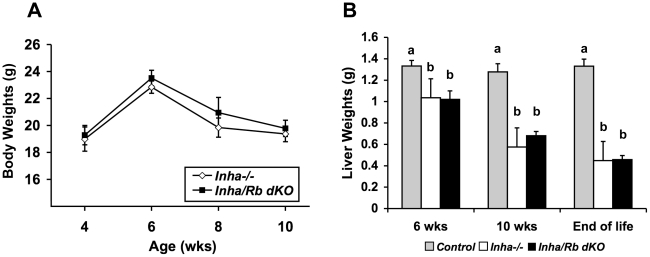
Double knockout of *Inha* and *Rb* in Sertoli cells does not significantly affect the activin-induced wasting syndrome. Body weights (A) and liver weights (B) are not significantly different between age-matched *Inha^−/−^* and *Inha/Rb* dKO mice (p>0.05). Control liver weights are shown for comparison. Different letters represent statistically different groups.

Disease progression was also monitored by examining other parameters. Tumor burden of the combined gonads was determined at 6 weeks, 10 weeks, and end of life. *Inha/Rb* dKO mice did not differ significantly from *Inha^−/−^* mice at every age examined (Figure 2B, p>0.05). Gonadal tumors in *Inha^−/−^* mice secrete an excess of activins, which signal through type 2A activin receptors (ACVR2A) to cause a cachexia wasting syndrome characterized by decreased body weights and liver weights [23], [24]. An examination of body weights from 4–10 weeks of age showed no significant differences between *Inha^−/−^* and *Inha/Rb* dKO (Figure 3A, p>0.05). Similarly, liver weights from 6 weeks, 10 weeks, and end of life were also not significantly different between these groups (Figure 3B, p>0.05).

Serum hormone levels were also measured in 10 week-old mice, due to their implications in disease progression [12], [13], [25]. No significant differences were detected between *Inha^−/−^* and *Inha/Rb* dKO levels of FSH, LH, or testosterone (T) (Table 1). The only significant difference detected was in estradiol (E2) levels, which were significantly decreased in *Inha/Rb* dKO mice as compared to *Inha^−/−^* mice (Table 1). Although prior studies have indicated that loss of estrogen receptors in *Inha*-null tumors prevented early tumorigenesis and death [25], we saw no functional consequence of this decreased estradiol level as disease progression in *Inha/Rb* dKO mice did not differ significantly from *Inha^−/−^* mice, as outlined previously.

**Table 1:** Serum Hormone Levels for 10 wk-old Males.

Genotype	FSH (ng/ml)N.S., *	LH (ng/ml)N.S.	T (ng/dl)N.S.	E2 (pg/ml)
*Inha^+/−^ Rb^+/−^* (n = 5)	15.7±1.7	0.08±0.03	46.7±8.2	5.2±0.2^a^
*Inha^−/−^* (n = 9)	26.8±3.6	0.14±0.04	60.1±9.8	83.7±15.0^b^
*Inha/Rb* dKO (n = 9)	23.0±2.6	0.16±0.03	90.4±22.1	25.2±14.2^a^

Values are means ± standard errors.

Statistically different values by Tukey-Kramer HSD are represented by different letters in superscript (a vs. b, p<0.05).

N.S., Not significant by one-way ANOVA (p>0.05).

*, FSH values of *Inha^+/−^ Rb^+/−^* vs. *Inha^−/−^* and *Inha^+/−^ Rb^+/−^* vs. *Inha/Rb* dKO are significantly different by one-tailed *t* test (p<0.05).

### Loss of *Inha* accelerates the progressive Sertoli cell dysfunction observed in Sertoli cell-specific RB deficient mice

**Figure 4:**
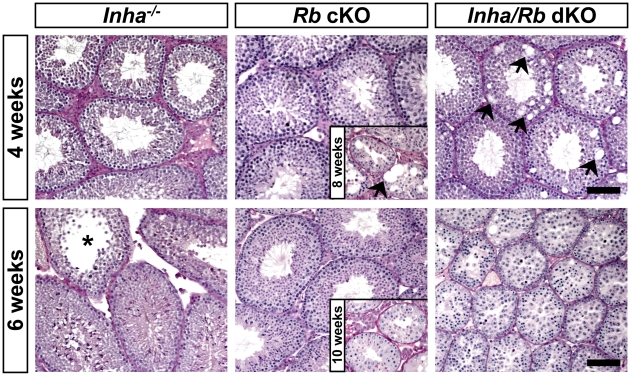
Seminiferous tubules in *Inha/Rb* dKO mice show an acceleration of the Sertoli cell dysfunction seen in *Rb* cKO mice. Histological examination of the seminiferous epithelium of 4 week-old *Inha^−/−^* (*top left*), *Rb* cKO (*top middle*), and *Inha/Rb* dKO (*top right*) mice reveals the presence of normally maturing germ cells in all genotypes, but 4 week-old *Inha/Rb* dKO Sertoli cells contain vacuoles (*top right*, arrows), a sign of Sertoli cell dysfunction that is seen in *Rb* cKO mice at older ages (*top middle, inset*). Areas of 6 week-old *Inha^−/−^* and *Rb* cKO testes that contain seminiferous tubules exhibit all stages of spermatogenesis (*bottom left and middle, respectively*), although some *Inha^−/−^* tubules are showing signs of dysfunction, such as loss of elongating spermatids (*bottom left*, asterisk). In contrast, areas of 6 week-old *Inha/Rb* dKO testes that contain seminiferous tubules show signs of extreme Sertoli cell dysfunction, such as loss of seminiferous tubular lumen, decreased tubular width, and loss of maturing germ cells (*bottom right*), that resemble advanced stages of infertility progression in *Rb* cKO mice (*bottom middle, inset*). All panels are captured at 100× magnification; scale bars, 100 µm.

Since we could not discern any striking differences between *Inha^−/−^* and *Inha/Rb* dKO mice using the criteria described above, we sought to examine mice from younger stages of disease progression. Histology of 4 week-old and 6 week-old *Inha/Rb* dKO testes did not show increased tumorigenesis when compared to age-matched *Inha^−/−^* testes (data not shown). However, when we examined the non-tumorigenic areas that contained intact seminiferous tubules, we observed an acceleration of the progressive infertility phenotype of the *Rb* cKO mice [6]. In *Rb* cKO mice, histological signs of Sertoli cell dysfunction, such as vacuolization, are not observed until roughly 8 weeks of age (Figure 4, *top row middle inset*) [6]. Loss of the tubular lumen, severely decreased tubular widths, and severe loss of germ cells generally occurs at 10 weeks of age (Figure 4, *bottom row middle inset*) [6]. In *Inha/Rb* dKO testes, there were prominent signs of vacuolization (Figure 4, *top row right panel*, arrows) in 4 week-old tubules that were not observed in either *Inha^−/−^* or *Rb* cKO testes (Figure 4, *top row*). However, 4 week-old *Inha^−/−^*, *Rb* cKO, and *Inha/Rb* dKO testes showed similar germ cell compositions (Figure 4, *top row*). In 6 week-old *Inha/Rb* dKO testes, there were signs of severe Sertoli cell dysfunction, such as loss of tubular lumens, decreased tubular widths, and loss of advanced germ cells, that resembled much older *Rb* cKO mice (Figure 4, *bottom row*). Examination of 6 week-old *Inha^−/−^* and *Rb* cKO testes revealed multiple tubules with full complements of germ cells, including elongating spermatids, and intact tubular widths and lumens (Figure 4, *bottom row*). Some *Inha^−/−^* tubules showed loss of elongating spermatids (Figure 4, asterisk), but overall, the seminiferous tubules of 6 week-old *Inha^−/−^* and *Rb* cKO mice showed far less dysfunction than 6 week-old *Inha/Rb* dKO mice.

**Figure 5:**
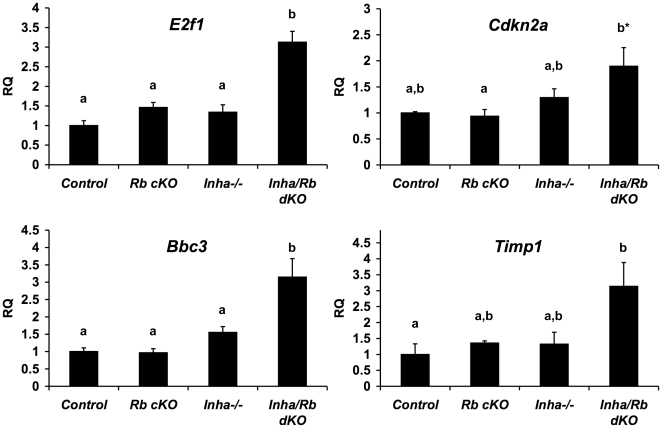
Select gene changes in *Inha/Rb* dKO mice as examined by quantitative PCR. *Inha/Rb* dKO levels of *E2f1* (*top left*), *Bbc3* (*bottom left*), and *Timp1* (*bottom right*) are all significantly different from control by Tukey's HSD (a vs. b, p<0.05). Although *Inha/Rb* dKO levels of *Cdkn2a* (*top right*) are not significantly different from control by Tukey's HSD, they are significantly different by student's t-test (asterisk, p<0.05).

To investigate the cause of the accelerated Sertoli cell dysfunction in *Inha/Rb* dKO mice, we explored changes in mRNA levels of genes that were implicated in the pathogenesis of the *Rb* cKO phenotype [6]. We used RNA from whole testes of 4 week-old mice that exhibited only minor histological differences from control mice to examine select gene changes. Genes related to *Rb* cKO apoptotic defects (*E2f1*, *Cdkn2a*, and *Bbc3/PUMA*) and differentiation defects (*Timp1*) were all significantly increased in *Inha/Rb* dKO testes as compared to control (Figure 5).

## Discussion

Whereas absence of p27 in *Inha* KO mice accelerates the formation of Sertoli cell tumors [15], our present studies indicate that RB plays a minor or redundant role in *Inha* KO tumorigenesis. By following the disease progression of *Inha/Rb* dKO mice as compared to *Inha^−/−^* mice, we determined that loss of RB does not significantly affect a number of parameters associated with *Inha* KO tumorigenesis, including survival, tumor burden, body weight, or liver weight. However, our findings do show that the progressive Sertoli dysfunction exhibited in *Rb* cKO mice [6] is accelerated by the additional loss of inhibin α.

**Figure 6:**
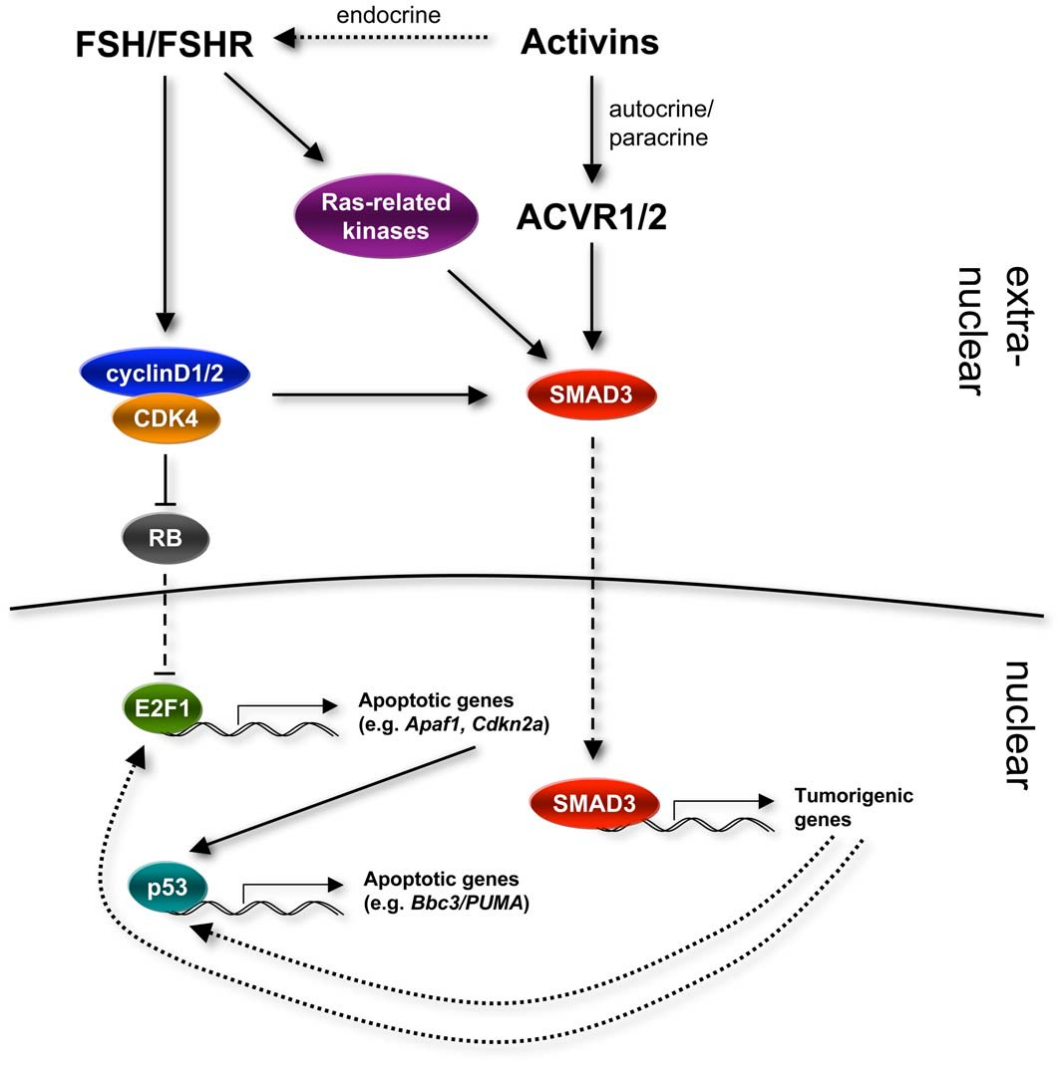
Proposed mechanism of Sertoli cell tumorigenesis in *Inha* ^−/−^ mice and acceleration of dysfunction in *Inha/Rb* dKO mice. Activins signal through type 1 and 2 activin receptors (ACVR1/2) to phosphorylate and activate SMAD3. Deletion of inhibin α not only leads to loss of competitive inhibition at the level of ACVR1/2 receptors, but it also leads to a pathological increase in activin levels. In our model, excess activin and FSH signaling synergistically activate SMAD3 tumorigenic pathways. Although the current studies suggest RB is not an important modifier of the inhibin α KO phenotype, we speculate that FSH mediates crosstalk between the RB pathway and inhibin α KO tumorigenic pathway by activating cyclin D1/2, which also modulates SMAD3 activity to affect tumor development and progression. We propose that SMAD3 activation synergizes with loss of RB in *Inha/Rb* dKO Sertoli cells thus resulting in the upregulation of E2F1- and p53-target genes. Solid line, direct interaction; dotted line, indirect interaction; dashed line, nuclear translocation.

Activin levels are pathologically increased in *Inha*-null mice [23] and are the major cause of tumorigenesis in these mice. This is suggested by studies in which decreased levels of free activins decreased tumor progression and disease severity [26]. Also, double knockout of *Inha* and *Smad3*, the activin-responsive transcription factor, resulted in attenuation of Sertoli cell tumorigenesis [20], [27]. These studies suggest that pathologically increased activins are in large part responsible for Sertoli cell tumorigenesis in *Inha*-null mice. Why is it that Sertoli cells react by becoming tumorigenic since the entire organism is exposed to increased activins in the bloodstream? Previous studies conducted in our lab indicated that gonadotropins, especially FSH, are essential for the progression of *Inha* KO tumorigenesis in male mice [11], [12]. Perhaps, it is the gonadotropin-responsiveness of Sertoli cells that makes them uniquely susceptible to pathological activin signaling. FSH signaling in Sertoli cells can activate the Ras-related mitogen activated signaling pathway involving ERK1 (mitogen-activated protein kinase 3) and ERK2 (mitogen-activated protein kinase 1), which subsequently upregulate cyclin D1 [28]. CDK4/cyclin D complexes and other Ras-related kinases modulate the phosphorylation of SMAD3 [29], [30], [31], and this modulation of SMAD3 causes its downstream signaling to change from tumor suppressive to oncogenic [29], [30]. We were very surprised at the minor effect of loss of RB on the disease progression in *Inha*-null mice. However, since our hypothesis was based on double knockout models that suggested that RB pathway components, specifically p27 and cyclin D2, modulate *Inha* KO tumorigenesis, we speculate that crosstalk between the RB and activin/FSH pathways were responsible for our previous results. In our model (Figure 6), the pathway of FSH signaling is crucial for establishing crosstalk between signaling of the components of the RB pathway of cell cycle control (cyclin D1/2) and activin signaling to result in tumorigenesis; however, retinoblastoma protein itself is not crucial for oncogenesis in this model. It will be important in the future to study the expression levels of activin pathway components and RB pathway components (*e.g.*, cyclin D1) in our *Rb*/*Inha* dKO mouse to further clarify if our model of *Inha* KO tumorigenesis is correct.

Retinoblastoma protein is a major determinant of Sertoli cell maturation, and mice with Sertoli cell-specific depletion of RB have progressively dysfunctional testes after puberty [6]. In light of this knowledge, it is quite interesting that deletion of *Rb* on an *Inha*-null background causes acceleration of the phenotype of progressive Sertoli cell dysfunction. This could be due to increased FSH signaling in *Inha*-null mice as FSH levels are not increased in *Rb* cKO mice until older ages [6]. Additionally, activation of SMAD3 in *Inha*-null Sertoli cells may cause an elevation of tumorigenic gene expression that may subsequently increase E2F1- and p53-target genes (Figure 6). We propose that these pathways converge to affect Sertoli cell function in *Inha/Rb* dKO mice.

Previously, we proposed that RB was important for Sertoli cell differentiation because of its interactions with androgen receptor [6]. The current studies raise the possibility that the pathological expression of cell cycle genes on an RB-deficient background are also directly related to Sertoli cell differentiation. Strikingly, we found that *Timp1*, a gene important to the inhibition of collagen-remodeling that is highly expressed in immature Sertoli cells, was upregulated in *Inha/Rb* dKO mice. *Timp1* was also highly upregulated in Sertoli cell-specific *Rb* cKO mice. The early upregulation of *Timp1* in *Inha/Rb* dKO testes as compared to *Rb* cKO testes suggests that differentiation genes like *Timp1* may be related to cell cycle pathways downstream of RB and SMAD3. Elucidation of our proposed model will be vital to better understanding the functions and regulation of the Sertoli cell.
